# The relationship between nature exposure and depression among Chinese prisoners: a moderated mediation model

**DOI:** 10.3389/fpsyg.2024.1252864

**Published:** 2024-02-21

**Authors:** Yuze Zeng, Qingqi Zhang, Jinglu Yan, Ke Qi, Ai Ma, Xiaoqian Liu, Junze Xiao

**Affiliations:** ^1^School of Criminal Justice, China University of Political Science and Law, Beijing, China; ^2^School of Sociology, China University of Political Science and Law, Beijing, China; ^3^Institute for Social Science Research, The University of Queensland, Brisbane, QLD, Australia; ^4^The Psychological Counseling Center, China University of Political Science and Law, Beijing, China

**Keywords:** nature exposure, depression, meaning in life, CU traits, moderated mediation model

## Abstract

**Aim:**

This study examined the association between self-reported nature exposure and depression among Chinese prisoners, as well as the mediating and moderating effects of meaning in life and callous-unemotional (CU) traits, respectively.

**Background:**

Prisoners are more likely to experience depression than any other mental illness. Exposure to nature has been proposed as a highly cost-effective method of treating their depressive symptoms. However, the mechanism underlying the link between nature exposure and depression among prisoners needs further investigation, as the findings may provide new insights into how to address depression in incarcerated populations.

**Method:**

Data were collected through a survey conducted in four prisons in southern China from April to May 2022. The participants were 574 prisoners who anonymously completed four questionnaires about nature exposure, meaning in life, depression, and CU traits.

**Results:**

The results show that: (1) meaning in life significantly mediates the association between nature exposure and depression, and (2) CU traits moderate the connection between nature exposure and meaning in life.

**Conclusion:**

The current study uncovered that prisoners who contact more with the natural environment have a higher meaning in life and lower depression, and individuals with higher CU traits can benefit more from nature exposure.

## 1 Introduction

Depression is distinguished by continuous sorrow, a loss of interest or enjoyment, and decreased energy (World Health Organization, 2017; Dattani et al., 2018). It is one of the greatest challenges facing the world (Abdu et al., 2018). Incarcerated people have a higher prevalence of depression than the general population, due to the uncontrolled, unfree, and threatening nature of the prison environment. A recent survey found that 17.9% of prisoners in Britain had depressive episode (Butcher et al., 2021). Another study found that 9–29% of inmates in the United States experience depression (Prins, 2014). In China, Geng et al. (2021), who recruited 1,484 male Chinese prisoners from Guangdong, discovered that the depression rate was 28.8%. Indeed, depression is the most common mental health disorder among prisoners (Johnson and Zlotnick, 2012), prison officials unfortunately often overlook it (Iversen et al., 2014).

Depression among inmates has a range of negative consequences that pose a significant threat to the prison management bureau. Generally, prisoners’ mental health problems are directly associated with various forms of deviant behavior, such as violence, suicide, and escape attempts (Fazel et al., 2016; Majekodunmi et al., 2017). Furthermore, higher levels of depressive symptoms impair prisoners’ social adaptation and increase the risk of future recidivism (Chang et al., 2015; Wallace and Wang, 2020). Studies conducted in China have also revealed imprisonment itself raises individuals’ depressive levels and that depressed prisoners have more acute suicidal thoughts and behaviors, particularly in Chinese female prisoners (Zhou et al., 2014; Zhong S. et al., 2019; Zheng et al., 2021). Therefore, identifying potential protective factors for depression among inmates is an important issue related to prison safety and the effectiveness of offender rehabilitation (Bezold et al., 2018).

Regarding the question of how to reduce depression among inmates, recent studies have shed light on the significance of the socio-physical factors of the prison environment (Moran, 2019; Moran and Turner, 2019), especially the pivotal role of the natural environment (e.g., forests). A growing number of studies demonstrated significant associations between nature exposure and reduced depressive symptoms among general adolescents, adults, and elders (Gidlow et al., 2016; Jia et al., 2016; Li et al., 2018). Some research has indicated that inmates could benefit from exposure to nature (Moran, 2019; Moran and Turner, 2019; Reddon and Durante, 2019); one empirical study demonstrated that indirect nature exposure can alleviate prisoners’ psychological issues, including depression (Li et al., 2021). Therefore, although nature exposure may play an important role in influencing prisoners’ depression, the mechanisms underlying the association between exposure to the natural environment and depression in this population require further investigation. Accordingly, this study examined whether nature exposure can reduce depression among inmates, considering the mediating and moderating effects of perceived meaning in life and callous-unemotional (CU) traits, respectively.

### 1.1 Nature exposure and depression among prisoners

Nature exposure is defined as how much an individual (group) has contact with natural environments (Bratman et al., 2019). It often occurs when one is outdoors, while exposure can also happen indoors in the presence of a window or indoor plants (Beute and de Kort, 2018). Stress reduction theory posits that people have an intrinsic need to commune with nature. Spending time in nature can trigger individuals’ parasympathetic nervous system and positive emotions, reducing their stress response and improving mental health (Silva et al., 2018; Han and Hyun, 2019; Norwood et al., 2019; Zhang et al., 2020). Accumulating empirical research indicates that being exposed to natural surroundings such as green areas (e.g., forests, grass, and trees) has numerous mental health benefits and positive effects for reducing depression (Silva et al., 2018; Helbich et al., 2019; Norwood et al., 2019; Zhang et al., 2020). Tomasi et al. (2020) suggested that frequent contact with nature during outdoor activities lessens depressive symptoms. Roberts and Helbich (2021) conducted a seven-day smartphone-based tracking study and further pointed out that exposure to green surroundings, both at home and on the road, has been linked to a reduction in depression. Some reviews and meta-analyses also confirmed the association of nature exposure and depressive disorder (Roberts et al., 2019; Jakstis and Fischer, 2021; Balanzá-Martínez and Cervera-Martínez, 2022). For example, a recent meta-analysis reported that short-term contact (10–90 min) with natural spaces is linked to a small decrease in depressive mood (Roberts et al., 2019). However, these findings have often been extrapolated from measurements of the general healthy population or clinical patients with depressive symptoms, whereas few studies have examined how nature exposure can benefit those who live in confined spaces, such as prisoners.

Prisons are well-known for being solitary settings where punishment takes precedence over rehabilitation (Reddon and Durante, 2019). Therefore, prisons are impoverished environments for inmates, who are usually restricted to rooms with inadequate ventilation and poor lighting. This situation threatens inmates’ mental health, leading to a high incidence of depression among incarcerated populations and difficulties in prison management. For example, at a physiological level, individuals in incarceration often suffer from vitamin D deficiency due to inadequate exposure to natural light (Pürner et al., 2018; De Leo et al., 2022). Vitamin D, beyond being a mere vitamin, also acts as a hormone and is directly associated with mental health disorders including depression (Casseb et al., 2019; Hernandez, 2023). Thus, the psychological rehabilitation of prisoners has begun to receive more consideration from the government. Psychologists use a range of methods, such as cognitive behavioral and mindfulness-based therapies, to reduce prisoners’ depression risk (Yoon et al., 2017); however, these techniques generally require adequate financial support and are highly dependent on trained professionals. Recently, nature contact has been recommended as a highly cost-effective tactic to improve prisoners’ psychological wellness. Some research suggests that activities such as gardening and looking at pictures of nature can reduce inmates’ risk of depression onset. Toews et al. (2018) found that a nature-based intervention could make incarcerated women feel happier, calmer, and less stressed, compared with pre-intervention levels. Furthermore, Li et al. (2021) found that the visibility of nature through windows positively affected life satisfaction, well-being, and distress tolerance, as well as reduced depression, in a sample of 326 male prisoners.

Therefore, based on the above findings, the following hypothesis is proposed:

*Hypothesis* 1 (H1): Nature exposure is negatively associated with depression among prisoners.

### 1.2 Mediating role of meaning in life

Further investigation is needed to clarify the mechanism underlying the relationship between nature exposure and depression among prisoners. Meaning in life refers to people’s conviction that their presence has value and recognition of a clear purpose and mission (Steger and Kashdan, 2013). As existential psychologist Rollo May stated, “Our relationships with nature support our sense of self, help us discover our purpose in life, and assist us manage our anxieties” (Softas-Nall and Woody, 2017, p. 241). Contact with nature may help people improve their skills, maintain good habits, and make better life plans (Nisbet et al., 2008; Cervinka et al., 2012), which are essential components of meaning in life. Moreover, nature exposure can enhance positive emotions and well-being (Li et al., 2021), which are important contributors to life significance. Some empirical evidence indicates that connectedness to nature may positively predict meaning in life (Howell et al., 2012; Aruta, 2021). Moreover, Hooker et al. (2022) found that nature-based experiences can improve meaning in life for veterans with posttraumatic stress disorder. Therefore, nature exposure can be assumed to significantly increase individuals’ meaning in life.

Meaning in life can negatively predict depression (Smedema Malonda and Franco Modenes, 2018; Dewitte et al., 2019; Parra, 2020; Sun et al., 2022). Meaning in life, according to logotherapy theory, has a long-lasting, positive impact on one’s mental health problems and subjective well-being (Frankl, 1992; Zhong M. et al., 2019). Empirical studies also demonstrate that the loss of personal significance is a major cause of depression (Steger and Dik, 2009; Vella-Brodrick, 2012; Steger et al., 2014) and that meaning in life can alleviate negative symptoms in depressed people.

Thus, in a prison environment, it is likely that nature exposure reduces depression by enhancing prisoners’ meaning in life. Imprisonment not only has a negative effect on individuals’ freedom and intimate relationships but also brings a profound existential crisis marked by a sense of meaninglessness, which is an important cause of depression (Van Ginneken, 2014; Vanhooren et al., 2017). Some prisoners describe incarceration as a feeling of “hitting rock bottom,” and often feel shocked by what they have done, losing pursuits and expectations of the future (Van Ginneken, 2014). In a survey conducted with 446 individuals, Addad (1987) found that meaning in life was significantly lower in incarcerated groups than in non-incarcerated groups and proved that an absence of meaning can positively predict distress in prison. In the face of meaninglessness and depression, prisoners require effective solutions. As mentioned above, nature exposure can provide prisoners with positive emotions and experiences of self-transcendence, connectedness, and security in a chaotic world (Howell et al., 2012). Conceivably, nature exposure is a catalyst for improving overall life significance (Hooker et al., 2022), which could mitigate depressive symptoms in prisoners. Accordingly, the following is hypothesized:

*Hypothesis* 2 (H2): Meaning in life mediates the effect of nature exposure on depression among prisoners.

### 1.3 Moderating effect of CU traits

Nature exposure can increase one’s sense of meaning in life (Hooker et al., 2022); however, even the same natural environment will not have an equal positive effect on all individuals. Extensive evidence has shown that the efficacy of exposure to nature greatly depends on individual personality traits (Takayama and Tsutsui, 2010; Song et al., 2013; Ambrey and Cartlidge, 2017; Feng et al., 2022). According to ecological systems theory, individual traits and the environment can interact to influence personal development (Bronfenbrenner, 1992). Furthermore, John et al. (2008) explained that personality traits affect what individuals see and how they interpret meaning in their surroundings, which can lead to differences in psychological outcomes.

In this study, we suggest CU traits may have a considerable moderating effect on the link between nature exposure and meaning in life among prisoners. CU traits refer to a stable personality tendency that cannot be easily changed, with core traits such as indifference to others, a lack of guilt, and low empathy (Frick, 2004). CU traits may be a unique and prominent personality pattern among criminal populations (Guo et al., 2017; Duindam et al., 2021) and are significantly associated with juveniles’ antisocial behavior, crime, and recidivism (Frick and White, 2008; Kimonis et al., 2016; Robertson et al., 2020; Sakki et al., 2023). For adults, CU traits are an affective component of psychopathy (Sakki et al., 2023); some studies also confirmed that individuals with high-level CU traits tend to have more antisocial and criminal behavior (Kahn et al., 2013; Keune et al., 2017; Falcón et al., 2021; Golmaryami et al., 2021; Lee and Choi, 2021). One previous study pointed out that, compared to prisoners with a greater sense of meaning in life, those with a lower sense of meaning in life displayed more negative world assumptions and less empathy for others (Vanhooren et al., 2016), which are typical characteristics of prisoners with higher CU traits. This indicates that CU traits are negatively relevant to meaning in life, and prisoners with higher CU traits may perceive less meaning in life.

Empirical research has suggested that people with high levels of neuroticism generally also have a higher degree of CU traits (Assary et al., 2014).These individuals often feel uneasy in natural settings because such environments may evoke profound reflections and musings (Tomasi et al., 2020). For prisoners with high CU traits, being exposed to a purely natural environment may provide a rare sense of awe, prompting them to reflect on past criminal behavior and the direction of their future life. Although this process is painful, it can help prisoners rediscover a sense of meaning in life. Thus, considering the interaction between personal traits and the environment (Tomasi et al., 2020), compared to those with lower CU traits, prisoners with higher CU traits may benefit more from nature exposure and experience greater improvement in their sense of meaning in life. Thus, the following hypothesis is proposed:

*Hypothesis* 3 (H3): The relationship between prisoners’ nature exposure and meaning in life is moderated by CU traits, and is stronger when prisoners’ CU traits are higher rather than lower.

### 1.4 The present study

**Figure 1:**
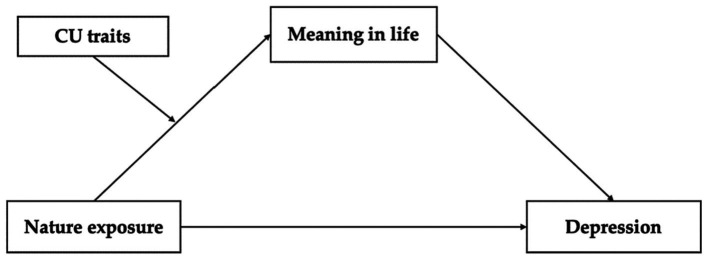
Moderated mediation model.

We created a moderated mediation model based on the aforementioned speculations to discover the connections of nature exposure, depression, meaning in life, and CU traits among prisoners. Specifically, we investigated the mediating role of meaning in life in the association between nature exposure and depression, and the moderating role of CU traits in the link between nature exposure and meaning in life. The hypothesized model is presented in Figure 1.

## 2 Methods

### 2.1 Participants

A survey was conducted in four prisons in southern China for data collection from April 2022 to May 2022. A total of 632 copies of the questionnaire were distributed to inmates, and the final sample consisted of 574 participants (women: 38.7%, men: 61.3%), corresponding to a 90.82% valid response rate. The questionnaires were completed anonymously. This study was approved by the Ethics Committee of the School of Sociology, China University of Political Science and Law. Table 1 shows participants’ demographic information.

**Table 1:** Demographic information of participants (*N* = 574).

Characteristic	Frequency	Percent (%)
Gender		
Man	352	61.32
Women	222	38.68
Age		
16–25	54	9.41
26–40	321	55.92
41–55	153	26.66
>55	46	8.01
Marital status		
Married	191	33.28
Single	262	45.64
Other*	121	21.08
Length of education		
0–6	103	17.94
7–9	305	53.14
10–12	70	12.20
>12	96	16.72
Have children		
Yes	313	54.53
No	261	45.47

*Widowed, separated, or divorced.

### 2.2 Measures

#### 2.2.1 Nature exposure

The Nature Exposure Scale (NES) constructed by Kamitsis and Francis (2013) was used to assess participants’ exposure to nature. The scale consists of four questions answered using a Likert scale ranging from 1 (not at all) to 5 (very). A higher total score indicates more significant levels of nature exposure (sample item: “How much do you notice the natural environments in your everyday life?”). In the present study, Cronbach’s α for the NES was 0.75, which was similar to a previous study (Kamitsis and Francis, 2013).

#### 2.2.2 Meaning in life

The study adopted the Meaning in Life Questionnaire originally constructed by Steger et al. (2006) and validated for a Chinese population by Gan (2010). This 10-item scale evaluates participants’ perceptions of the meaning of their lives (sample item: “My life has a clear sense of purpose”), with five questions each for the existence and pursuit of meaning. Responses are provided using a seven-point scale, and higher total scores reflect greater levels of meaning in life. In this study, Cronbach’s α for this scale was 0.86.

#### 2.2.3 Callous-unemotional traits

CU traits were evaluated using the Inventory of Callous-Unemotional Traits (ICU) developed by Essau et al. (2006). Specifically, this study used a revised Chinese version that has previously shown good reliability and validity (Yang and Huang, 2013). The ICU contains 24 questions (sample item: “I do not feel remorseful when I do something wrong”) with three subscales: uncaring, callous, and unemotional. Responses are provided using a four-point Likert scale ranging between 0 (not at all true) to 3 (absolutely true). Higher scores indicate higher levels of CU traits. In this study, Cronbach’s α was 0.80 for the overall scale and ranged from 0.76 to 0.81 for subscales. The corrected item-total correlation coefficients ranged from 0.25 to 0.58, with an average corrected item-total correlation of 0.40, and this result is generally consistent with previous studies (Cardinale and Marsh, 2020).

#### 2.2.4 Depression

In this study, depression was measured with a Chinese version of the Depression Anxiety Stress Scales-21 (DASS-21) subscale (Lovibond and Lovibond, 1995). This depression subscale has seven items (sample item: “I felt that I had nothing to look forward to”), which are rated four-point scale ranging from 0 (never) to 3 (frequently). Higher score reflect a higher tendency toward depression. In this study, Cronbach’s α for this subscale was 0.83.

#### 2.2.5 Control variables

Previous studies indicated that prisoners’ demographic characteristics could have an impact on their depression levels (Yang et al., 1995; Karel, 1997; Silverstein et al., 2017; Shen, 2020). Thus, participants’ gender, age, marital status, education level, and whether they had children were controlled for in this study. Gender was dummy coded as 1 for “men,” 2 for “women” and 3 for “other.” Regarding age, participants were categorized into four groups: 16–25, 26–40, 41–55, and over 55. Marital status was categorized as 1 for “single,” 2 for “married,” and 3 for “other.” Education level was determined by number of years of schooling completed, classified into four categories: 0–6, 7–9, 10–12, and over 12. Whether participants had children was dummy coded as 1 for “yes” and 0 for “no.”

### 2.3 Statistical analysis

For statistical analysis, SPSS 25.0 (IBM, Armonk, NY) was used for the common method deviation test, descriptive statistics, and Pearson correlation analyses. The hypotheses were tested using Hayes’s PROCESS macro (Hayes, 2013). Meaning in life was employed as a mediator between nature exposure and depression by using Model 4 of the PROCESS macro. The moderated mediating effect was analyzed using Model 7 of the PROCESS macro. With 95% confidence intervals, the mediation and moderated mediation effects were tested using a total of 5,000 bootstrap samples. Before the analyses, all quantitative variables were standardized.

## 3 Results

### 3.1 Common method variance test

As the present research data were collected based on self-reported measures, it may lead to common method bias. In order to mitigate the impact of biases on the study’s findings, this research employed anonymous inquiry and utilized reverse scoring for some items in the program control. To examine the influence of common method bias, the Harman single-factor test was conducted by exploratory factor analysis, with 45 items combined together. The results show there were nine factors with eigenvalues greater than 1; the first factor could be accountable for 16.74% of the variance, less than 40% (Podsakoff et al., 2003), indicating that there was no significant common method bias in this study.

### 3.2 Descriptive statistics and correlations

Table 2 shows the descriptive statistic results among the study variables. Table 3 shows the correlations between nature exposure, meaning in life, depression and CU traits. As hypothesized, nature exposure was positively connected to meaning in life (*r* = 0.33, *p* < 0.01) and was inversely correlated with depression (*r* = −0.30, *p* < 0.01). Meaning in life was negatively correlated with depression (*r* = −0.27, *p* < 0.01) and CU traits (*r* = −0.20, *p* < 0.01). CU traits was negatively correlated with nature exposure (*r* = −0.34, *p* < 0.01) and was positively with depression (*r* = 0.23, *p* < 0.01).

**Table 2:** Descriptive statistics among variables (*N* = 574).

	M	SD	Range	Number of items	Reliability	Range of corrected item total correlation	Average corrected item total correlation
1. Nature exposure	12.59	2.23	5 ~ 19	4	0.75	0.43 ~ 0.65	0.55
2. Meaning in life	47.99	10.70	10 ~ 70	10	0.86	0.51 ~ 0.69	0.62
3. CU traits	24.61	7.82	3 ~ 58	24	0.80	0.25 ~ 0.58	0.40
4. Depression	5.76	4.20	0 ~ 17	7	0.83	0.45 ~ 0.70	0.57

**Table 3:** Correlations among variables (*N* = 574).

	1	2	3	4
1. Nature exposure	(0.47)			
2. Meaning in life	0.33 ^**^	(0.38)		
3. CU traits	−0.34 ^**^	−0.20 ^**^	(0.37)	
4. Depression	−0.30 ^**^	−0.27 ^**^	0.23 **	(0.37)

^**^p < 0.01. Index in () is the multiple correlation between variables.

### 3.3 Mediation analyses

The mediating role of meaning in life in the relationship between nature exposure and depression was examined using Model 4 of the PROCESS macro (Hayes, 2013) after controlling for gender, age, marital status, education level, and having children. As Table 4 shows, nature exposure positively predicted meaning in life (*β* = 0.31, *SE* = 0.04, *p* < 0.001) and negatively predicted depression (*β* = −0.21, *SE* = 0.04, *p* < 0.001). Meaning in life negatively predicted depression (*β* = −0.19, *SE* = 0.04, *p* < 0.001). These analyses demonstrate that meaning in life partially mediates the relationship between exposure to nature and depressive symptoms (indirect effect = −0.05, 95%*CI* [−0.093, −0.029]). Generally, this model accounted for 22.03% of the total effect, which supported Hypothesis 1 and Hypothesis 2.

**Table 4:** The mediation effect of meaning in life (*N* = 574).

Outcome	Predictors	*β*	*SE*	*p*	LLCI	ULCI
Meaning in life	Nature exposure	0.31	0.04	<0.001	0.227	0.393
	*R*^2^ = 0.11, *F* = 12.03, *p* < 0.001					
Depression	Nature exposure	−0.21	0.04	<0.001	−0.294	−0.122
	Meaning in life	−0.19	0.04	<0.001	−0.272	−0.108
		*R*^2^ = 0.14, *F* = 13.03, *p* < 0.001					

LLCI, lower limit of 95% confidence interval; ULCI, upper limit of 95% confidence interval.

### 3.4 Moderated mediation analyses

**Figure 2:**
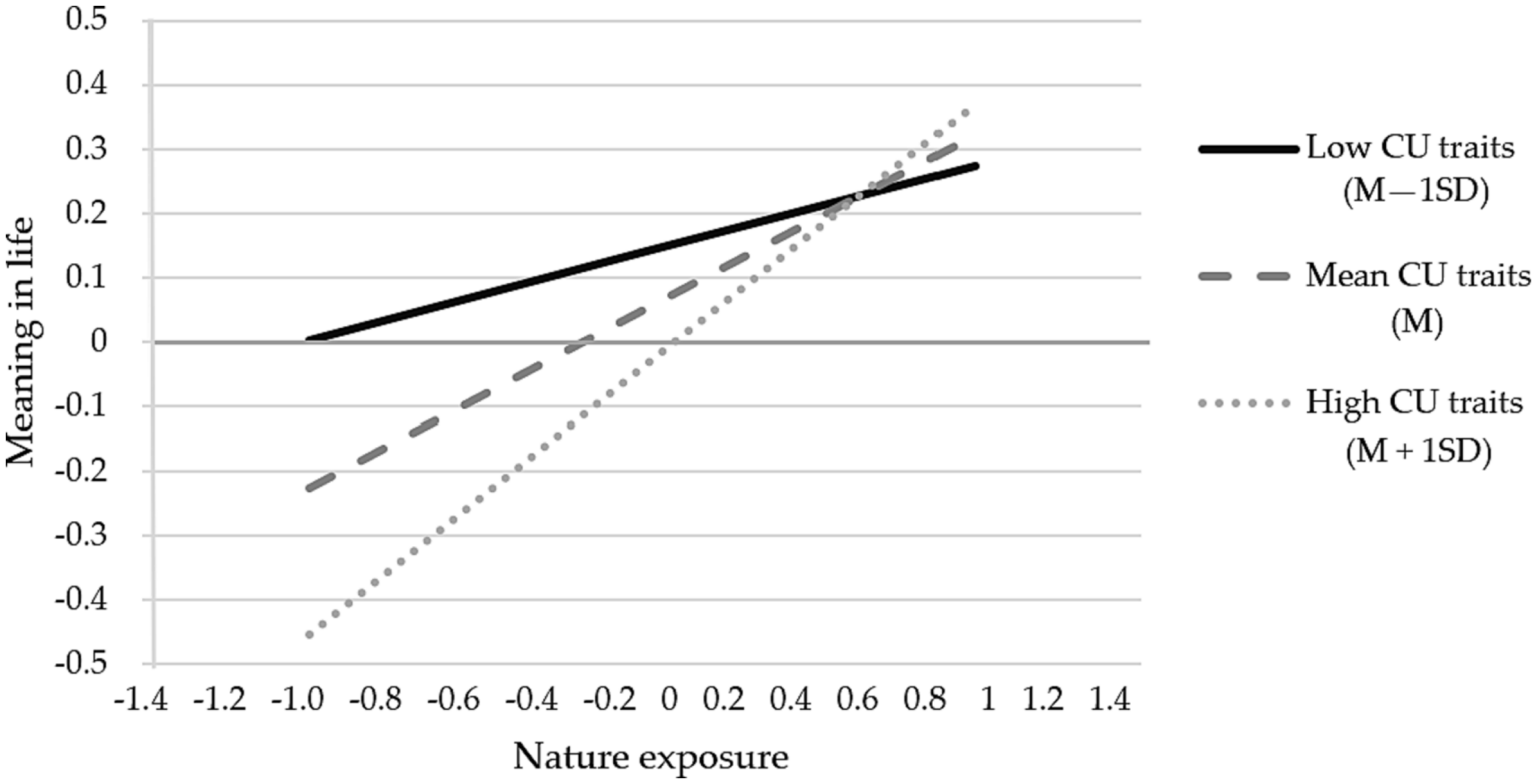
Moderating effect of CU traits on the correlation between nature exposure and meaning in life (*N* = 574).

Model 7 of the PROCESS macro was used to test whether CU traits moderate the mediating effect of meaning in life in the relationship between nature exposure and depression. The demographic variables noted above were controlled for in all analyses. As Table 5 shows, nature exposure positively predicted meaning in life (*β* = 0.27, *SE* = 0.04, *p* < 0.001), and this association was moderated by CU traits (β = 0.14, *SE* = 0.04, *p* < 0.001). As the simple slope test in Figure 2 indicates, nature exposure had a stronger positive impact on meaning in life (β_simple_ = 0.41, *SE* = 0.06, *p* < 0.001) for prisoners with higher CU traits (1 SD above the mean) than for prisoners with lower CU traits (β_simple_ = 0.14, *SE* = 0.06, *p* = 0.025).

**Table 5:** The moderated mediation effect of cu traits (*N* = 574).

Outcome	Predictors	*β*	*SE*	*p*	LLCI	ULCI
Meaning in life	Nature exposure	0.27	0.04	<0.001	0.189	0.358
	CU traits	−0.09	0.04	0.032	−0.177	−0.008
	Nature exposure × CU traits	0.14	0.04	<0.001	0.061	0.221
	*R*^2^ = 0.14, *F* = 11.48, *p* < 0.001					
Depression	Nature exposure	−0.21	0.04	<0.001	−0.294	−0.122
	Meaning in life	−0.19	0.04	<0.001	−0.272	−0.108
	*R*^2^ = 0.14, *F* = 13.03, *p* < 0.001					

LLCI, lower limit of 95% confidence interval; ULCI, upper limit of 95% confidence interval.

According to our results (see Table 6), the indirect effect of nature exposure on depression via meaning in life was substantially stronger for inmates with higher CU traits (*β* = −0.08, *SE* = 0.02, 95% *CI* [−0.123, −0.040]) than for those with lower CU traits (*β* = −0.03, *SE* = 0.01, 95% *CI* [−0.055, −0.003]). In addition, the bias-corrected 95% confidence interval for the moderated mediation index did not contain zero (Table 7), further demonstrating that CU traits moderated the indirect effects of nature exposure on depression via meaning in life, confirming Hypothesis 3.

**Table 6:** Conditional indirect effect of nature exposure on depression through meaning in life at different levels of CU traits (*N* = 574).

CU traits	Indirect effect	Boot *SE*	Boot LLCI	Boot ULCI
Low (Mean − 1 *SD*)	−0.03	0.01	−0.055	−0.003
Mean	−0.05	0.02	−0.083	−0.025
High (Mean + 1 *SD*)	−0.08	0.02	−0.123	−0.040

LLCI, lower limit of 95% confidence interval; ULCI, upper limit of 95% confidence interval. Number of bootstrap samples: 5000.

**Table 7:** Index of moderated mediation (*N* = 574).

	Index	Boot *SE*	Boot LLCI	Boot ULCI
CU traits	−0.03	0.01	−0.049	−0.010

Number of bootstrap samples: 5000.

## 4 Discussion

Although substantial empirical evidence supports the benefits of nature exposure for mental health, further research is needed. Based on stress reduction theory, this study proposes a novel model to explain how nature exposure can reduce depression in prisoners, by examining the previously unexplored mediating effect of meaning in life and moderating role of CU traits. The results indicate that nature exposure reduces depression among prisoners by improving their sense of meaning in life. Moreover, the correlation between nature exposure and meaning in life is moderated by CU traits. Specifically, prisoners with higher CU traits benefit more from nature exposure, with greater improvements to their sense of meaning in life. These findings identify a novel pathway for reducing prisoners’ depressive symptoms, especially those with high CU traits.

### 4.1 Nature exposure and depression among prisoners

First, in line with prior research, we found a negative relationship between nature exposure and depression (McEachan et al., 2016; Silva et al., 2018; Helbich et al., 2019; Norwood et al., 2019; Tomasi et al., 2020; Zhang et al., 2020; Roberts and Helbich, 2021). While some previous studies have found that the relationship between nature exposure and depression is not always negatively correlated, and certain elements within the natural environment may even have a negative impact on mental health. For instance, Zhang et al. (2019) found a positive correlation between long-term exposure to outdoor PM10 air pollution and depression, and Fowler et al. (2013) found no correlation between experiencing natural disasters and depression. However, our study does confirm the protective effect of natural exposure against depression in the prison context. The results support a prior study conducted among prisoners that found a link between viewing nature through prison windows and reduced depression risk. Stress reduction theory suggests that exposure to a natural environment, such as vegetation, can quickly stimulate positive thoughts that eliminate negative emotions, thereby reducing the body’s stress response and lowering the likelihood of depression (Markevych et al., 2017). On a physiological level, natural environments can elicit positive emotional responses such as pleasure and calmness, which help to reduce physiological stress indicators (Hartig et al., 2014) and then reduce depressive levels. Reddon and Durante (2018) pointed out that individuals’ nature exposure is a continuum from nature exposure sufficiency to insufficiency. Compared with nature exposure insufficiency, nature exposure sufficiency can help people reinvigorated from health problems, including arthritis, dementia, and depression. Moreover, the feeling of “being away” in natural settings, as a core positive outcome of nature exposure, has been further substantiated in recent literature, highlighting its significance in providing a mental break from stressors, which is especially beneficial for individuals in confined environments (Bratman et al., 2012). For prisoners, incarceration isolates them from the outside world, which leads to depression, anxiety, and many other mental health problems. Psychological therapy and physical activity are recommended to alleviate these problems; however, they require more material and professional resources. Comparatively, nature exposure may be a more practical and affordable option. Since 2008, England’s prison system has offered horticultural and environmental activities for prisoners through the “Greener on the Outside of Prisons” therapeutic program. According to a qualitative study, from 2008 to 2015, this program helped improve the mental health of over 4,500 prisoners in northwest England (Farrier et al., 2019). In sum, it is clear that nature exposure protects prisoners from depression, confirming Hypothesis 1.

### 4.2 Mediating role of meaning in life

This study revealed that meaning in life mediated the association between nature exposure and depression in inmates, confirmed Hypothesis 2. On the one hand, nature exposure was positively linked to meaning in life. Various reasons may lead to this phenomenon. First, natural environments provide a space of peace and restoration where inmates can engage in emotional regulation (Naor and Mayseless, 2021) and evoke feelings of happiness, satisfaction, and well-being; these are important factors that influence inmates’ sense of meaning in life. Second, nature exposure can make individuals feel connected with nature (Richardson et al., 2016), prompting them to engage in deep thinking, including reflection on personal behavior and goals (Kaplan, 1995; Herzog et al., 1997). Thus, following nature exposure, reflecting on past antisocial behaviors can help prisoners reject criminal thinking and restore the value of individual existence. Furthermore, another possible reason that nature exposure increases the sense of meaning in life is that the esthetic experience of the natural environment is an activity meaningful in itself. It can provide a sense of intrinsic satisfaction, which is a significant component of the sense of meaning in life (Howell et al., 2012).

On the other hand, meaning in life was found to be negatively related to depression, as prisoners with a greater sense of meaning in life reported fewer depressive symptoms. Numerous research has shown that meaninglessness is related to psychological illnesses, including depression (Hedayati and Mahmoud, 2014; Dewitte et al., 2019; Sun et al., 2022). Theorists and researchers concur unequivocally that a meaningful existence is essential for humans (Ryff and Singer, 1998; Deci and Ryan, 2000), and meaning in life might be an important protective factor against depression (Steger et al., 2009). For prisoners, searching for meaning in life can help them overcome the frustrations of imprisonment and create plans for a new life in the future, which can relieve their depressive symptoms. Thus, nature exposure has an indirect effect on depression via meaning in life, and this study further revealed an essential mediator of the relationship between nature exposure and depression.

### 4.3 Moderating effect of CU traits

This study found that CU traits moderate the connection between nature exposure and meaning in life. Specifically, nature exposure showed a greater impact on improving meaning in life in prisoners with high CU traits than those with low CU traits. Previous studies confirmed that people with higher CU traits generally had a poor childhood environment and grew up with harsh family education styles (McDonald et al., 2011; Waller et al., 2018), which may lead to a diminished perception of meaning in life (Baumrind, 1978; Maschi et al., 2011). Thus, if therapeutic intervention can improve individuals’ meaning in life, the outcome will be naturally more significant in people exhibiting higher rather than lower CU traits. Some research has also indicated that CU traits may impact treatment responses. Specifically, people with higher CU traits have shown greater treatment benefits in reducing their negative beliefs and behaviors (Farmer et al., 2015; Mattos et al., 2017; Bansal et al., 2019). These results are congruent with the ecological systems theory proposed by Bronfenbrenne, which posits that the natural environment and personality traits can interact to influence individual development. For prisoners with higher CU traits, the natural environment can elicit a sense of awe that is hard to appear in their lives and prompts them to reflect on their past criminal behavior, providing good opportunity to reflect deeply and restore a sense of meaning in life. Another possible explanation is that prisoners with higher CU traits, due to their limitations in emotion and empathy, may have poorer interpersonal relationships in their daily lives, experiencing more social isolation (Fanti et al., 2013). Therefore, the tranquility of the natural environment and the escape from the oppressive prison setting may provide these individuals with a unique opportunity for respite and relaxation that is unattainable in their everyday lives (Kaplan, 1995). This contrast could make the experience of nature contact more novel and restorative for individuals with higher CU traits, leading to a more significant enhancement of their meaning in life. Therefore, compared to those with lower CU traits, prisoners with higher CU traits benefit more from nature exposure and experience grater improvement to their sense of meaning in life, which supports Hypothesis 3. Thus, this study provides a new direction of life’s significance and mental health interventions for prisoners, especially those with higher CU traits.

### 4.4 Practical contributions

This study’s findings can provide significant insights for prison management. First, we discovered that the mental health benefits of nature exposure could expand to the criminal population. This suggests that prisons should organize more outdoor activities in green spaces or encourage prisoners to pay more attention to the natural scenery to protect them from outcomes such as self-harm and suicide caused by depression. For example, correctional officers could guide inmates to perform activities such as meditation and physical exercise in forests or grassy areas, providing them more opportunities to connect with nature. Alternatively, the integration of biophilic design within prison architecture, infusing natural elements into the built environment, can also enhance the inmates’ daily exposure to nature (Kellert et al., 2011). Second, the mediation effect found in this study emphasizes the important protective function of meaning in life against depression. Thus, in the context of prison education and rehabilitation, adding a curriculum to improve prisoners’ sense of meaning in life is an effective strategy to reduce depression. Furthermore, psychological therapeutic techniques related to existentialism may be particularly beneficial as they can help individuals find meaning and purpose in life (Vos et al., 2015). Third, the relationship between nature exposure and meaning in life is moderated by CU traits. Therefore, we recommend that mental health intervention plans be formulated according to personality traits. Specifically, for individuals with higher CU traits, prison administrators should provide them with more opportunities to observe green scenery and be close to nature, guiding them to pay more attention to the natural environments and learn to engage in deep thought. For individuals with lower CU traits, prison administrators can still adopt traditional methods to improve their sense of meaning in life and decrease depressive symptoms. Finally, the natural scenery should not be too “hard” (e.g., watching auto racing in a green environment), as “soft” scenery, such as plants, sunrises, clouds, and snowflakes, have better effects (Herzog et al., 1997). Only focusing on these “soft” sceneries requires little effort and can provide sufficient room for reflection.

### 4.5 Limitations

Although this study deepens the current understanding of the link between nature exposure and depression among Chinese prisoners, it still has several limitations. First, we utilized a cross-sectional method for data collection; thus, causality cannot be determined. Future studies can use experimental methods to validate a causal link, and a longitudinal design can be used to examine whether the results change over time. Second, in the future, more comprehensive and targeted measurements about nature exposure should be made for groups such as inmates living in restricted environments. This study, only investigates the general degree of nature exposure among inmates. New tools should also consider the quality of nature exposure experiences, the duration and frequency of nature exposure, and individual preferences for specific natural settings. These more detailed measurements can give us a better understanding of the natural contact of inmates. Third, by employing a variety of research methodologies, it is possible to assess whether different forms of natural exposure elicit distinct impacts on depression. We examined exclusively the correlation between previous levels of nature exposure and depression in this study. Further research could investigate whether there is a distinction in the effect size produced by direct outdoor nature exposure, exposure to indoor green plants, and viewing nature images or videos. Additionally, it would be beneficial to identify more economical methods of nature exposure. Fourth, this study used self-report questionnaires, and some inmates may disguise their true beliefs due to certain concerns. In follow-up studies, different data sources, such as physiological indicators or other people’s evaluations (e.g., correctional officers, peers) should be collected to verify the reliability of the results. Fifth, future research should consider a broader range of variables. We recognize that this study did not delve into the more profound roles that demographic factors, such as gender, having children, and education, may play in relation to nature exposure, meaning in life, depression, and callous-unemotional traits. While these were controlled for, their potential moderators were not explored. Future research could investigate these relationships more thoroughly. Sixth, all study participants were from southern China; however, the climate, natural environment, and prison management vary across regions and have different effects on prisoners’ depression. Thus, future studies should collect data across a wider range of areas in China.

## 5 Conclusion

Overall, the current study demonstrated that nature exposure negatively correlates prisoners’ depression, and meaning in life mediated the relationship between nature exposure and depression. Furthermore, the positive association between nature exposure and meaning in life was moderated by CU traits. These results imply that prisoners who contact more with the natural environment have a higher meaning in life and lower depression, and individuals with higher CU traits can benefit more from nature exposure.

## Data availability statement

The raw data supporting the conclusions of this article will be made available by the authors, without undue reservation.

## Ethics statement

The studies involving humans were approved by The Ethics Committee of the School of Sociology, China University of Political Science and Law. The studies were conducted in accordance with the local legislation and institutional requirements. The participants provided their written informed consent to participate in this study.

## Author contributions

YZ, JX, and QZ conceived the idea. JY and KQ designed the questionnaires and collected the data. QZ was responsible for data curation. YZ and JX wrote the first version of the manuscript. XL and JX evaluated and revised the text. XL, JX, and AM supervised the project and secured funding. All authors contributed to the article and approved the submitted version.
